# Self-Tolerance of Vascular Tissues Is Broken Down by Vascular Dendritic Cells in Response to Systemic Inflammation to Initiate Regional Autoinflammation

**DOI:** 10.3389/fimmu.2022.823853

**Published:** 2022-01-26

**Authors:** Li Sun, Wenjie Zhang, Lin Zhao, Yanfang Zhao, Fengge Wang, Andrew M. Lew, Yuekang Xu

**Affiliations:** ^1^ Anhui Provincial Key Laboratory for Conservation and Exploitation of Biological Resources, College of Life Science, Anhui Normal University, Wuhu, China; ^2^ The Walter & Eliza Hall Institute of Medical Research and Dept of Microbiology & Immunology, University of Melbourne, Parkville, VIC, Australia

**Keywords:** vascular tissues, regional immunity, dendritic cells, immunotolerance, autoinflammation

## Abstract

The correlation of infections with vascular autoinflammatory diseases such as vasculitis and atherosclerosis has been long recognized, and progressive inflammation with the formation of tertiary lymphoid organs in arterial adventitia intensively studied, the immunological basis of the nondiseased vasculatures that predispose to subsequent vascular autoimmunity during inflammation, however, is not well characterized. Here, we investigated the vascular immunity *in situ* of steady-state C57BL/6 mice and found that healthy vascular tissues contained a comprehensive set of immune cells with relatively higher proportion of innate components than lymphoid organs. Notably, a complete set of dendritic cell (DC) subsets was observed with monocyte-derived DCs (moDCs) constituting a major proportion; this is in contrast to moDCs being considered rare in the steady state. Interestingly, these vascular DCs constitutively expressed more suppressive factors with cDC1 for PD-L1 and moDCs for IL-10; this is concordant with the inhibitive phenotype of T cells in normal vascular tissues. The immunotolerant state of the vascular tissues, however, was readily eroded by systemic inflammation, demonstrated by the upregulation of proinflammatory cytokines and enhanced antigen presentation by vascular DCs to activate both cellular and humoral immunity *in situ*, which ultimately led to vascular destruction. Different vascular DC subsets elicited selective effects: moDCs were potent cytokine producers and B-cell activators, whereas cDCs, particularly, cDC1, were efficient at presenting antigens to stimulate T cells. Together, we unveil regional immunological features of vascular tissues to explain their dual facets under physiological versus pathological conditions for the better understanding and treatment of cardiovascular autoinflammation.

## Introduction

As the first line of defense against the invasion of foreign pathogens, leukocytes are often present at peripheral barrier tissues, including the skin, gastrointestinal tract, and respiratory tract. In response to infectious challenge, innate immune cells respond quickly often overcoming the invaders; however, in case the pathogens persist, innate cells alert the adaptive immune cells, which are most abundant in secondary lymphoid organs (SLOs) such as the spleen, lymph nodes, and Peyer’s patches. Beyond these tissues, immune cells may also reside in, albeit in rare numbers, deep nonbarrier tissues such as adipose, meninges, uterus, and cardiovascular tissues. Indeed, evidence has accumulated that vascular tissues might possess immunological potency (1). Anatomically, large vascular walls are surrounded by vasa vasorum (small blood vessels that provides nutrients and oxygen where simple diffusion from the lumen of the large vessel itself is not sufficient) and lymphatic vessels that provide routes of leucocyte trafficking and interstitial fluid drainage (2). Additionally, leukocytes may gather in the blood vessels in expandable adventitial cuffs *via* interstitial fluid flow constitutively or chemotactically (3). Such above pathways could possibly constitute their own unique immune network and avail leucocytic infiltration within vascular walls as early as the first year of childhood (4).

The presence of immune cells in large- and medium-size blood vessel walls in the steady state suggests that they may be autodestructive during disease states. Clinically, vascular tissues have long been documented as the important sites of various autoinflammatory diseases including giant cell vasculitis (5) and atherosclerosis (6) with involvement of resident immune cells. Recent studies have suggested that local innate immunity and immunometabolism of dendritic cells (DCs) and T cells have emerged as new actors during atherogenesis (7, 8). DCs are key to bridging between innate and adaptive immunity. They can be broadly divided into several functional subsets with distinct phenotypes and functions. pDCs (CD11c^interm^B220^+^), for example, secrete high levels of type 1 IFN; cDC1 (CD11c^hi^CD11b^−^CD8^+^/CD103^+^) are efficient at priming CD8^+^T cells, and cDC2 (CD11c^hi^CD11b^hi^ CD8^−^/CD103^−^) are potent at priming CD4^+^T cells (9). In addition, monocyte-derived DCs (moDCs; CD11c^interm^CD11b^hi^) are rare during the steady state but rapidly abound during inflammation and are potent in promoting Th1 and Th17 immunity (10).

Subjected to multiple stimulation by vascular risk factors such as smoking and obesity, oxidized low-density lipoprotein (oxLDL) can activate DCs to recruit mononuclear immune cells and gradually form a lymphoid structure named vascular-associated lymphoid tissue or arterial tertiary lymphoid organ (ATLO) (11). Interestingly, in patients with atherosclerosis, DCs were significantly more abundant in plaque areas when surrounded by ATLOs (12) and can play both atherogenic and atheroprotective roles in various disease settings (13, 14). A CCL17-secreting DC subset, for example, was identified in atherosclerotic lesions at the aortic root but not in the healthy vascular walls; this subset promoted atherosclerosis by limiting regional regulatory T-cell (Treg) expansion and increasing plaque burden (15). On the other hand, depletion of CD103^+^DCs in Flt3^−/−^Ldlr^−/−^ mice led to heavier plaque burden, decreased aortic Treg numbers, limited interleukin-10 (IL-10) secretion, increased production of interferon-γ (IFN-γ) and tumor necrosis factor-α (TNF-α) without significant alterations in lipid levels (16), indicating an immunosuppressive role for this DC subset in atherosclerosis. Unfortunately, most of these studies on the role of DCs in vasculitic diseases lacked specificity, as they were performed *via* depleting pan DC marker CD11c that could also be expressed on other cell type such as macrophage and activated T cells during inflammation (17). Although recent studies of some transcription factors such as IRF4 (18) and Zbtb46 (19), allow more specific identification of the role of DCs in atherosclerosis, what they assessed was the impact of systemic DCs on atherosclerosis, leaving open the contribution of local vascular DCs.

The differentiation and functions of the DCs during diseases, like many other immune cells, heavily depend on the local microenvironment where they are developed at steady state. Microanatomic niches with various auxiliary cells in the tissues regulate the life cycles of locally resident immune cells (20). Therefore, it is likely that the stromal niches in vascular tissues (which are different from that of SLOs) might confer unique immunological properties on local immune cells and thus affect disease progression. Adventitial cuffs, for example, are rich in collagen, extracellular matrix components, and mesenchymal, fibroblast-like adventitial stromal cells (ASCs) that express unique cytokine profiles to support distinct tissue immunity *via* DCs and Tregs (3). Therefore, identification of the constitutive immunological features of vascular tissues would be important in understanding pathogenesis and hence therapeutic pathways.

## Materials and Methods

### Mice

Specific pathogen-free (SPF) C57BL/6 and OT-II mice, aged 10 weeks were purchased from Hefei BoYuan Laboratory Animal Ltd., Hefei, China, and housed in a SPF environment in the Anhui Normal University Animal Facilities. OT-II mice are transgenic for a T-cell receptor that recognizes ovalbumin presented on MHCII (OVA^329-337^-IA^b^). All procedures conducted on mice were in accordance with the conditions approved by the Institutional Animal Care and Use Committee of Anhui Normal University.

### Single Cell Suspensions and Flow Cytometry

Aortic single cells and splenocytes were prepared through previous methods with minor modifications (21).

#### Aortic Vascular Tissue

Mice were anesthetized by injecting i.p. 500 µl of 5% chloral hydrate in PBS, and their vasculature was perfused by cardiac puncture with PBS containing 20 U/ml of heparin extensively to remove blood from all vessels, before single cell suspensions were prepared from aortic segments, including aortic sinus with valve or aortic arch and thoracic segments using previous methods with minor modifications. In brief, after full removal of the perivascular fat and cardiac muscle tissues, using microscissors under a dissecting microscope, single cell suspensions from 2 aortas were prepared by incubation with 2 ml enzyme mixture containing 400 U/ml collagenase I, 120 U/ml collagenase XI, 60 U/ml hyaluronidase, and 60 U/ml DNase1 in modified Dulbecco’s PBS with calcium and magnesium for 60 min at 37°C with gentle shaking. A cell suspension was obtained by mashing the vascular tissues through a 70-µm strainer.

#### Spleen

Splenic single cell suspensions were prepared by treatment with 0.5 μg/ml of collagenase type II for 55 min and 2 mM of EDTA for another 5 min. Erythrocytes were then removed by exposure to red blood cell lysis buffer. Single cell suspensions of aortic single cells and splenocytes prepared as above were stained with indicated antibodies in the excess amounts of Fc receptor blocking antibody for 30 min on ice and added propidium iodide (1 μg/ml) at the final wash to stain dead cells. Flow-cytometric analysis was performed with flow cytometer (FACS Melody, BD Biosciences, San Jose, CA, USA). Flow Jo software was used to analyze the data.

### Phagocytosis Assay

Phagocytic activity of vascular DCs was estimated by their uptake of soluble antigens followed by FACS analysis as described before (22). Briefly, C57BL/6 mice were injected *i.v.* with OVA-FITC. After 6 h, aortic single cells and splenocytes were isolated and stained with color-conjugated antibodies against CD45, CD64, CD11c, CD11b, MHCII, B220, and XCR1 for 30 min on ice. Flow-cytometric analysis was performed with flow cytometer (FACS Melody, BD Biosciences, San Jose, CA, USA). Flow Jo software was used to analyze the data.

### DC Cytokine Assay

Cytokines of vascular DCs were estimated by intracellular staining as described previously (23). Briefly, C57BL/6 mice were injected *i.v.* with 5 µg lipopolysaccharide (LPS) (L4391, Sigma, St. Louis, MO, USA). After 12 h, aortic single cells and splenocytes were isolated from mice and stimulated with GolgiSTOP Protein Transport Inhibitor (51-2092KZ, BD) for 4–6 h at 37°C under 5% CO_2_ environment. Cells were washed and then stained with antibodies against CD45, CD64, CD11c, CD11b, MHCII, B220, and XCR1. Cells were fixed, perforated, and stained intracellularly with IL-12/IL-23 p40, IL-1β, and IL-10 for 30 min on ice. Flow-cytometric analysis was performed with BD FACS Melody flow cytometer.

### T-Cell Proliferation Assay

T-cell proliferation was estimated by Ki67 and CFSE dilution *via* FACS analysis.

#### T-Cell Proliferation *In Situ* (Ki67 Assay)

C57BL/6 mice were injected *i.v.* with 20 µg LPS. After 48 h, aortic single cells and splenocytes were isolated and stained with FACS antibodies against CD45, CD11b, CD19, CD3, CD4, and CD8 for 30 min on ice. After, cells were fixed by the fixation and permeabilization solution (BD, 51-2090KZ, San Diego, US), and perforated by Perm Buffer III (558050, BD, San Diego, USA). Then, cells were stained intracellularly with Ki67 for 30 min on ice. Flow-cytometric analysis was performed with BD FACS Melody flow cytometer.

#### OVA-Specific T-Cell Proliferation

*(1) OVA injection into OT-II mice:* 200 µg OVA protein in saline were injected *i.v.* into OT-II mice. After 3 days, vascular tissues and spleen were taken. Ki67 expression of CD4^+^T cells were measured. *(2) CFSE assay:* freshly isolated OT-II CD4^+^ T cells were incubated with 5 µM CFSE in 1 ml PBS per 1 × 10^7^ cells for 10 min at 37°C and checked by flow cytometry for effective staining. In total, 6 × 10^6^ CFSE-labeled cells in 200 µl PBS were then *i.v.* injected into CD45.1 mouse. After 24 h, mice were *i.v.* injected with OVA protein (60 µg) and LPS (20 µg) in 200 µL PBS. After 4 days, aortic single cells and splenocytes were isolated and stained with FACS antibodies against CD45.1, CD45.2, and CD4 for 30 min on ice. Proliferation of OT-II-derived CD4^+^T cells was determined by flow cytometry using the CFSE dilution assay.

#### Cross-Presentation Assay *In Vivo*

A total of 100 µl OVA (500 µg) or PBS were mixed with equal volume of complete Freund’s adjuvant (CFA) (Sigma), vortexed for 2 min, and injected *i.p.* into WT mice. One week later, the mice were restimulated with the same procedure. After 4 days, 200 µl OVA (1.5 mg) or PBS were *i.v* injected into the mice, and the OVA antigen-specific activation of CD8^+^T cells in terms of proliferation and IFN-g secretion were measured by intracellular staining of Ki67 and IFN-g using protocols and reagents outlined above 3 days later.

### T-Cell Differentiation Assay

The differentiation of T cells was performed using standard methods as described before (24). Briefly, C57BL/6 mice were injected *i.v.* with 20 µg LPS. After 48 h, aortic single cells and splenocytes were isolated and stimulated with cell-stimulation cocktail plus protein transport inhibitors (00-4975-93, Invitrogen, San Diego, CA, USA) for 4–6 h at 37°C under 5% CO_2_ environment. After that, cells were washed and then stained with CD45, CD11b, CD19, CD3, CD4, and CD8. Cells were then fixed, perfornated, and stained intracellularly with IFN-γ, IL-17A, IL-10, and TGF-β1 or cells were fixed, perforated by the Foxp3 buffer set (560409, BD, San Diego, CA, USA), and then stained intracellularly with Foxp3 (560403, BD). Flow-cytometric analysis was performed with BD FACS Melody flow cytometer.

### Histopathology and Immunohistochemistry

C57BL/6 mice were injected *i.v.* with 20 µg LPS. After 48 h, vascular tissues and spleen were harvested and fixed with 4% paraformaldehyde in phosphate buffer. Paraffin-embedded samples were sectioned at 5 µm, and then stained with hematoxylin-eosin (HE) and Weigert (resorcin-fuschin) staining for histological analysis, rabbit anti-mouse CD45 (GB11066, Servicebio, Wuhan, China) for leukocytes, rabbit antimouse CD3 (14-0114-85, Invitrogen) for T cells, and rabbit anti-mouse CD11c (GB11059, Servicebio) for DCs. The nucleus was stained by DAPI. Different histological stains were observed using an Olympus microscope (BX-UCB, Olympus Corporation, Tokyo, Japan), and elastic lamellae disruptions were counted (×40 objective).

### Immunofluorescence

C57BL/6 mice were injected *i.v.* with 20 µg LPS. After 48 h, vascular tissues and spleen were harvested and snap-frozen in OCT compound (Sakura Finetek Denmark Aps, Brøndby, Denmark). Serial cryosections of 5 µm were cut along the vascular tissues and spleen, perfused with PBS, fixed by 4% paraformaldehyde overnight, and then stained with rat anti-mouse Foxp3 (14-5773-82, Invitrogen) and Goat-anti-rat IgG (H+L) (Alexa Fluor 488, A-11006, Invitrogen) or rabbit anti-mouse CD11c (GB11059, Servicebio), Armenian hamster anti-mouse CD3ϵ antibody (100301, Biolegend, San Diego, CA, USA), Goat anti-Hamster IgG (H+L) (Alexa Fluor 594, A-21113, Invitrogen), and Goat-anti-rabbit IgG (H+L) (Alexa Fluor 488, A31627, Invitrogen). The nucleus was stained by DAPI. Fluorescence images were captured by microscope (DMi8, Leica, Wetzlar, Germany). The intensity of fluorescence was evaluated with the ImageJ software as previously described

### Image Quantification

To count infiltrating cells, the areas of infiltration were selected manually and a customized macro for counting cells were run, generating an automatic cell count (25).

### RNA Extraction and Quantitative Real-Time Polymerase Chain Reaction

RNA extraction and quantitative real-time polymerase chain reactions were performed using standard methods as described previously (26). Briefly, total RNA was extracted from sorted DCs and transcribed into cDNA (PrimeScript™ RT reagent Kit with gDNA Eraser, TaKaRa, Beijing, China) according to the manufacturer’s instructions. Real-time qPCR with SYBR Green detection (SYBR Green Master Mix, SYBR^®^ Premix Ex Taq™II, TaKaRa, Beijing, China) was performed to measure RNA expression using a real-time qPCR detection system (CFX96, Bio-Rad, Singapore, Singapore). Analysis was performed using CFX Manager Software (Bio-Rad). Data were expressed relative to *β-actin* as an internal control. The primers used were as follows: *BAFF*-forward (TGC TAC TCG GCT GGC ATC GC), *BAFF*-reverse (GCG CGG GCT CCG TTT CTC AT); *ARRIL*-forward (GAC CCT GGT GGG TCT AGT GA), *ARRIL*-reverse (GTA GGA GCT GAG GCA TGA GG), and *β-Actin*-forward (AGC CAT GTA CGT AGC CAT CC), *β-actin*-reverse (TCC CTC TCA GCT GTG GTG GTG AA) as a housekeeping control.

### Statistical Analysis

Data were presented as mean ± SEM and analyzed using Prism (Graph Pad Software). Comparisons of experimental groups were analyzed by unpaired two-tailed Student’s *t*-test. The *p* < 0.05 was considered statistically significant.

## Results

### Comprehensive Immune Cell Composition in Vascular Tissues

**Figure 1:**
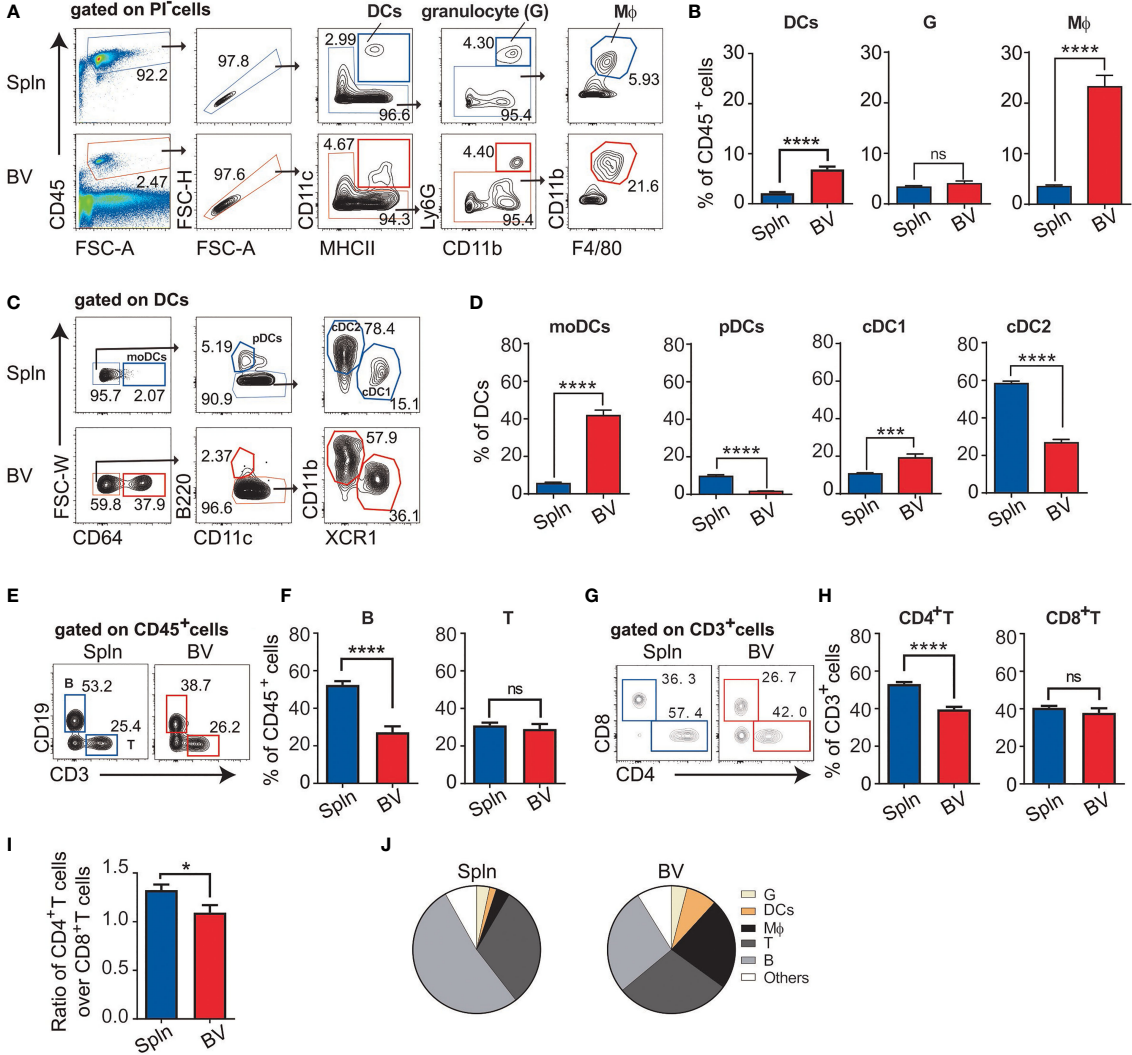
Profile of immune cell composition in vascular tissues. Cell suspensions from aortic blood vascular tissues (BV) and spleens (Spln) of healthy/noninflamed C57BL/6 mice were stained with indicated Abs for FACS analysis. Plots and graphs showed the gating strategy **(A)** and the proportion **(B)** of DCs, granulocytes, and macrophages; the gating strategy **(C)** and the proportion **(D)** of DC subsets; the gating strategy **(E)** and the proportion of B and T cells **(F)**; and the gating strategy **(G)** and the proportion **(H)** of T cell subsets, as well as the ratio **(I)** of CD4^+^T cells over CD8^+^T cells in BV and Spln. **(J)** Pie charts showing the proportion of different immune cells in BV and Spln. Shown here are representative plots of at least three independent experiments performed. The data are shown as mean ± SEM (*n* = 6–43 mice) and analyzed using Student’s *t*-test. ^*^ *p* < 0.05; ^***^ *p* < 0.001; ^****^ *p* < 0.0001. ns, no significant difference.

To comprehensively examine leukocyte composition within the vasculature, we compared vascular tissues with that of a canonical lymphoid organ (spleen) from steady-state C57BL/6 mice. DCs were identified as CD45^+^CD11c^+^MHCII^+^; granulocytes as CD45^+^CD11c^lo to neg^MHCII^− to +^Ly6G^hi^CD11b^+^; and macrophages as CD45^+^CD11c^lo to neg^MHCII^− to +^Ly6G^lo to neg^CD11b^− to +^F4/80^+^, respectively (**Figure 1A**). Compared with spleen, the proportion of DCs and macrophages within the immune cells were higher in the aortic blood vascular (BV) tissues (**Figure 1B**) whereas granulocytes were similar in these two organs. Of note, DCs in the vascular tissues consisted of all the main DC subsets (**Figure 1C**), in which the proportions of pDCs and cDC2 were lower; whereas, moDCs and cDC1 were higher than those of spleens (**Figure 1D**). Even though moDCs are relatively rare in the steady state (10, 27, 28), we found that the predominant vascular DC type was moDCs; whereas, it is the rarest DC type in the spleen (**Figure 1D**). In addition to the innate myeloid cells, we also investigated the adaptive immune components in the vascular tissues including B cells as CD45^+^CD19^+^ and T cells as CD45^+^CD3^+^ (**Figure 1E**). We found that the proportion of vascular B cells was lower than their splenic counterparts, whereas the proportion of vascular T cells was similar (**Figure 1F**). However, within the subsets of T cells, the percentage of vascular CD4^+^ and CD8^+^T cells was respectively lower and similar to those of the spleen (**Figures 1G, H**). Hence, the ratio of CD4^+^T:CD8^+^T in vascular tissues was lower (**Figure 1I**). Collectively, we identified a comprehensive set of immune cells in the vascular tissues but with varying proportions. In particular, the proportions of vascular DCs (about 3-fold increase) and macrophages (>5-fold increase) were relatively enriched compared with their splenic counterparts (**Figure 1J**).

### Vascular DCs Have an Immature and Tolerogenic Phenotype

**Figure 2:**
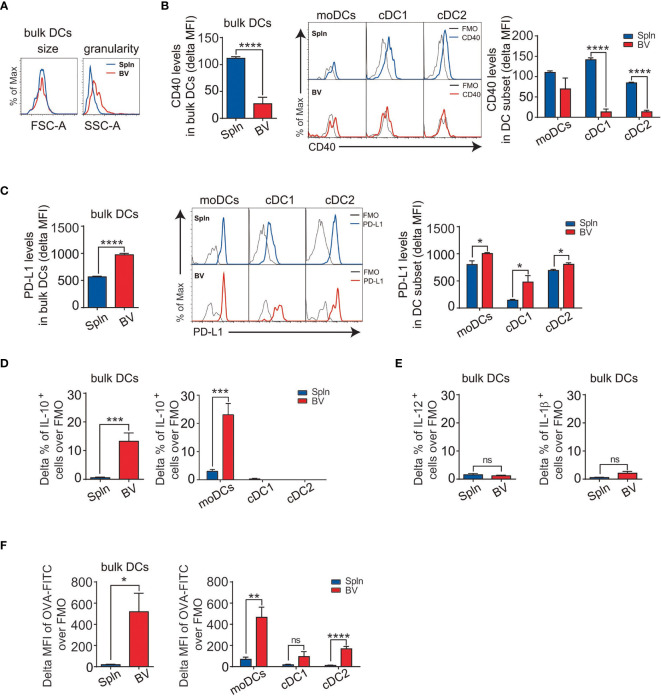
Immature DCs with suppressive activities and superior phagocytotic capacity in vascular tissues at steady state. Tissue single-cell suspensions from BV and Spln of healthy mice were prepared and gated for FACS analysis as in **Figure 1**. Plots and graphs show the size and granularity **(A)**, delta MFI of CD40 **(B)**, delta MFI of PD-L1 **(C)** expression, and delta percentage of IL-10 **(D)** in bulk DCs and their subsets, as well as IL-12/IL-1β **(E)** in bulk DCs. Alternatively, mice were injected with OVA-FITC for 6 h before the delta MFI of OVA-FITC in bulk DCs and their subsets **(F)** were analyzed. Shown here are representative plots of experiments with at least three biological replicates. The data are shown as mean ± SEM (*n* = 3–9 mice) and analyzed using Student’s *t*-test. ^*^ *p* < 0.05; ^**^ *p* < 0.01; ^***^ *p* < 0.001; ^****^ *p* < 0.0001. ns, no significant difference.

We foreshadowed that the relatively higher percentage of DCs in vascular tissues may be indicative of their importance in controlling local adaptive immunity. Therefore, we focused on this cell type first. Morphologically, the vascular DCs had similar size to that of splenic DCs, but they seemed to be more granular, as evidenced by the side scatter measurements in FACS analysis (**Figure 2A**). Phenotypically, vascular DCs, especially cDCs including cDC1 and cDC2, had generally lower expression of costimulatory molecule CD40 than that of splenic DCs (**Figure 2B**), indicating an immature DC phenotype. Due to their extreme scarcity in the vascular tissues [(29) and our data], we did not further characterize pDC subset in this study.

The relatively immature phenotype of vascular DCs is suggestive of tolerogenic potential in the steady state. Consistently, we found that vascular DCs had overall a higher expression of the coinhibitory molecule PD-L1 than that of splenic DCs (**Figure 2C**). This was represented by all three DC subsets, with cDC1 demonstrating the highest difference (**Figure 2C**). To further confirm the suppressive nature of vascular DCs, we next measured their intracellular levels of cytokines and surprisingly found that they constitutively secreted IL-10 at levels higher than that of splenic DCs (**Figure 2D**). Interestingly, this secretion of IL-10 could be solely attributed to moDCs, as there was little and no significant difference between splenic and vascular cDC1 and cDC2 subsets (**Figure 2D**). In contrast to the immunosuppressive cytokine, there was little difference in levels of proinflammatory cytokines (IL-12 and IL-1β between splenic and vascular DCs (**Figure 2E**).

Immature DCs prompted greater phagocytic and uptake capacity, and self-tolerance is associated with the constitutive capture and presentation of self-antigens to the immune system (30). As vascular DCs displayed an immature phenotype, we investigated their capacity for antigen uptake. We found that vascular DCs demonstrated much better capacity to uptake the soluble antigen than their splenic counterparts (**Figure 2F**). Furthermore, this stronger uptake of antigen by vascular DCs was more conspicuous in moDC than cDC subset (**Figure 2F**).

### Suppressed Phenotypes of Vascular T Cells at Steady State Agree With the Immaturity of Vascular DCs

**Figure 3:**
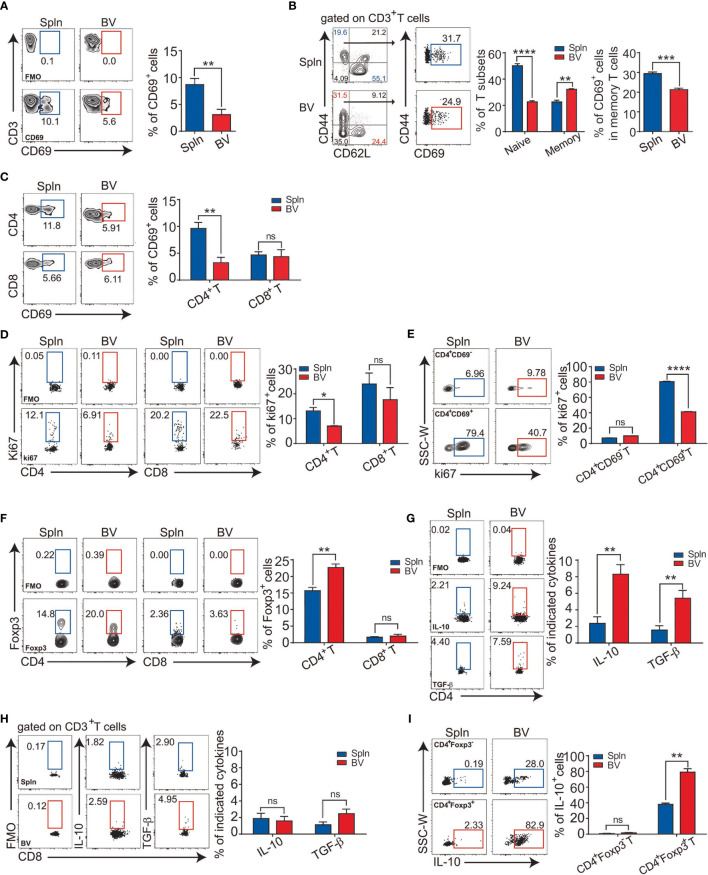
Immunologically suppressed phenotypes of vascular T cells at steady state. Tissue single-cell suspensions from BV and Spln of healthy mice were prepared and gated for FACS analysis as in **Figure 1**. Plots and bar graphs show the percentage of CD69 in T cells **(A)**, their naïve vs. effector fractions **(B)**, and T-cell subsets **(C)**; percentage of cells positive for Ki67 **(D)**, and the proportion of Ki67 in CD4^+^CD69^−^ and CD4^+^CD69^+^T cells (**E**); Foxp3 **(F)** in CD4^+^ and CD8^+^T cells; cytokine contents of IL-10/TGF-β1 in CD4^+^ **(G)** and CD8^+^T cells **(H)**, as wells as IL-10 in CD4^+^Foxp3^−^ and CD4^+^Foxp3^+^T cells **(I)**. Shown here are representative plots of experiments with at least three biological replicates. The data are shown as mean ± SEM and analyzed using Student’s *t*-test. ^*^ *p* < 0.05; ^**^ *p* < 0.01; ^***^ *p* < 0.001; ^****^ *p* < 0.0001. ns, no significant difference.

To verify the functional outcomes of the suppressive factors including surface molecules and soluble cytokines from the vascular DCs to local adaptive immunity, we investigated the activation state of the T cells in the vascular tissues *in situ*. We found that the percentage of the early activated, CD69^+^ vascular T cells were half that of splenic T cells (**Figure 3A**). In addition, we found that although there were less naïve T cells (CD62L^+^CD44^−^) and more memory T cells (CD62L^−^CD44^+^) in vascular tissues than spleens, the vascular memory T cells had significantly less expression of T-cell activation marker CD69, indicating a more quiescent state of the vascular resident T-cell population in comparison with splenic tissues (**Figure 3B**). Furthermore, at the subset level, the lower CD69 expression in vascular T cells was mainly attributed to CD4^+^T cells (**Figure 3C**). Consistently, we found that the percentage of proliferating vascular CD4^+^T cells *in situ* was lower than that of spleens whereas there was no difference for CD8^+^T cells (**Figure 3D**). Moreover, within CD4^+^T-cell fraction, we found that the percentage of Ki67 on CD4^+^CD69^+^ T cells were much higher than that of CD4^+^CD69^−^ T cells (**Figure 3E**), indicating proliferation was mainly from the activated cells. Interestingly, concordant with the constitutively higher levels of the IL-10 in vascular DCs, a higher proportion of Treg (CD4^+^Foxp3^+^) cells in vascular tissues was detected (**Figures 2D**, **3F**). Consistent with the above findings, the proportion of IL-10 and TGF-β1 producing CD4^+^T cells were also found to be higher in vascular tissues than spleens (**Figure 3G**) but not so in the case of CD8^+^T cells (**Figure 3H**). Notably, we observed that the IL-10 was mostly derived from CD4^+^Foxp3^+^ Tregs, whereas CD4^+^Foxp3^−^ T cells produced little, if any, IL-10. Furthermore, vascular Tregs produced more IL-10 than their splenic counterpart on a single-cell basis (**Figure 3I**). Collectively, one parsimonious conclusion would be that vascular DCs and CD4^+^T cells *in situ* maintain a tolerogenic milieu during the steady state.

### Vascular moDCs Have an Outstanding Capacity to Upregulate Proinflammatory Cytokine During Acute Systemic Inflammation

**Figure 4:**
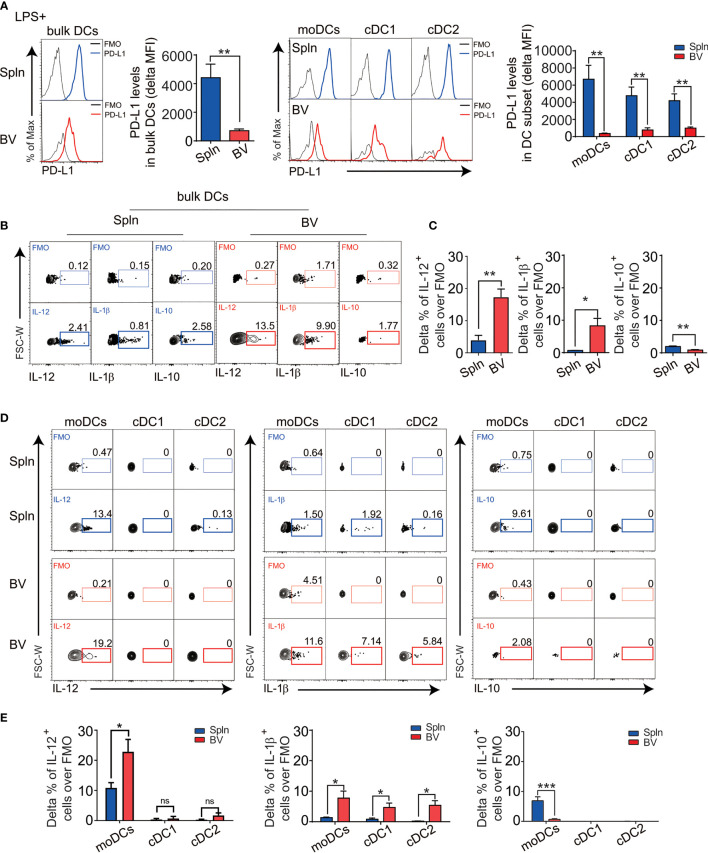
The responses of DC in vascular tissues and spleen during inflammation. Mice were injected *i.v.* with 5 µg LPS for 12 h before their BV and Spln were taken. FACS plots and bar graphs show the delta MFI of PD-L1 in bulk DCs and their subsets **(A)** and the indicated cytokine contents in bulk DCs **(B, C)** and their subsets **(D, E)**. Shown here are representative plots of experiments with at least four biological replicates. The data are shown as mean ± SEM (*n* = 4–8 mice) and analyzed using Student’s *t*-test. ^*^ *p* < 0.05; ^**^ *p* < 0.01; ^***^ *p* < 0.001. ns, no significant difference.

Next, we asked whether the tolerogenicity of the vascular tissues persisted following inflammatory stimuli or was readily broken down as occurred in many inflammatory vascular diseases like vasculitis and atherosclerosis. Healthy C57BL/6 wt mice were injected with bacterial lipopolysaccharide (LPS) to elicit acute systemic inflammation. We found that granulocytes were rapidly accumulated in the vascular tissues following LPS administration with a similar proportional increase to that of the spleen (**Figure S1**), indicating indistinguishable impact of the systemic inflammation on the two tissues. However, DCs, especially, moDCs, and their downstream T effector cells, are increased in the vascular tissues (**Figure S1**), suggesting their higher sensitivity to the inflammatory challenge. Remarkably, we found that systemic LPS-downregulated PD-L1 in vascular DCs more dramatically than that in splenic DCs on both moDC and cDC1/2 subsets (**Figure 4A**), even though PD-L1 expression was constitutively higher in vascular DCs at steady state (**Figure 2C**).

In striking contrast to the steady state, during inflammation, there was about 3–10-fold increase in numbers of vascular DCs secreting polarizing cytokines IL-12 (Th1) and IL-1b (Th17), but less anti-inflammatory cytokine IL-10 (**Figures 4B, C**), suggesting a proinflammatory conversion of vascular DCs in response to pathogen invasion. Interestingly, this increase in IL-12 and the decrease in IL-10 could be solely attributed to moDCs, whereas the increase in IL-1b could be contributed by all DC subsets (**Figures 4D, E**). Consistently, we found these vascular DCs express more TLR4, the pattern recognition receptor for LPS, mostly in moDC subset (**Figure S2**).

### Vascular cDC1 Are Unique in Activating T Cells *In Situ* Within Vascular Tissues During Inflammation

**Figure 5:**
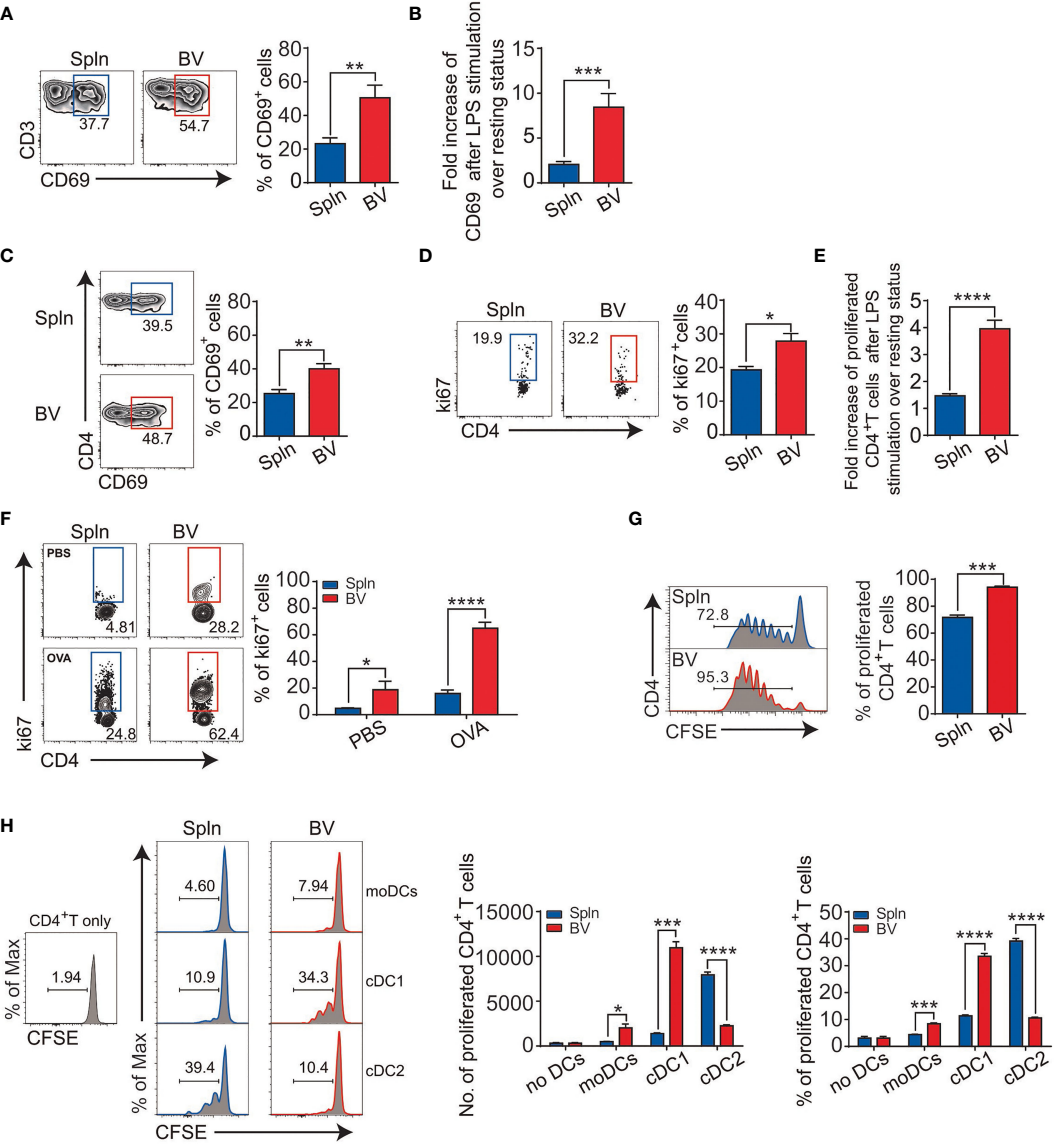
Enhanced CD4^+^T-cell activation and antigen presentation in vascular tissues during inflammation. Mice were *i.v.* injected with LPS before FACS analysis of the proportion **(A)** and fold increase **(B)** of CD69 in bulk T cells; percentage of CD69 **(C)**; proliferated Ki67^+^ cells **(D)**; and their fold increase **(E)** in CD4^+^T-cell subset. Alternatively, OT-II mice were *i.v.* injected with 200 µg OVA or PBS with LPS for 72 h before FACS analysis of proliferated CD4^+^T cells **(F)**. Purified CD4^+^T cells from OT-II mice were labeled with CFSE and injected into CD45.1 mice before being immunized with OVA plus LPS for FACS analysis of proliferated CD4^+^T cells **(G)**. Shown here are experiments with at least three biological replicates with error bars as mean ± SEM (*n* = 3–8 mice). Sorted DCs from CD45.2 mice following LPS administration were cocultured with CFSE-labeled CD4^+^T cells from OT-II mice in the presence of OVA for 3 days before the proliferation of CD4^+^T cells were measured by FACS **(H)**. Shown here are one representative data from two independent experiments performed. ^*^ *p* < 0.05; ^**^ *p* < 0.01; ^***^ *p* < 0.001; ^****^ *p* < 0.0001.

**Figure 6:**
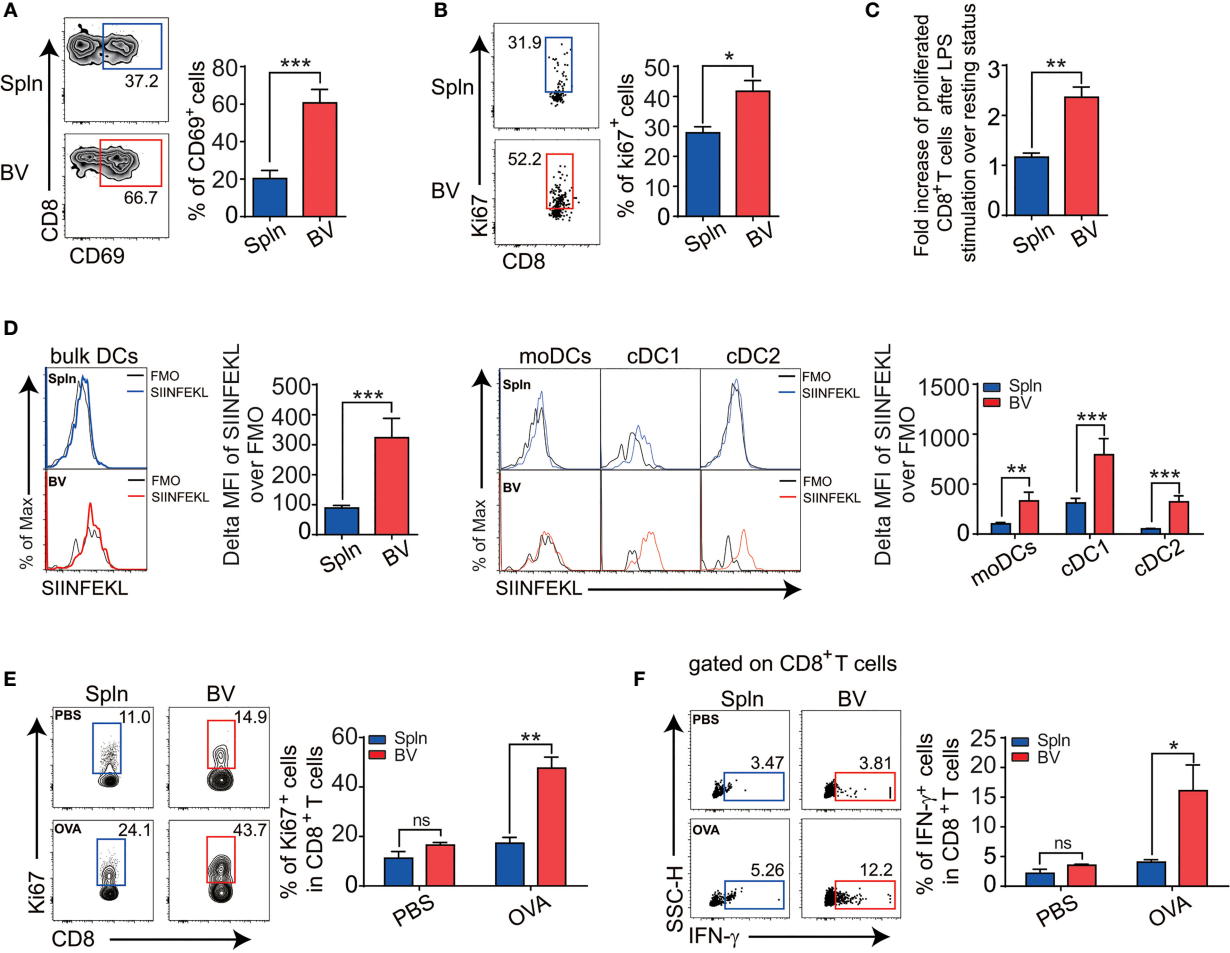
Enhanced CD8^+^T activation and cross-presentation in vascular tissues during inflammation. Mice were *i.v.* injected with LPS before FACS analysis of the proportion of CD69 **(A)**, cell positive for Ki67 **(B)**, and fold increase of proliferation after LPS stimulation over the steady state **(C)** in tissue CD8^+^ T cells. Alternatively, mice were *i.v.* injected with 1.5 mg OVA with 5 µg LPS for 12 h before the delta MFI of SIINFEKL in bulk DCs and DC subsets from two tissues examined by FACS **(D)**. WT mice were primed with OVA in the presence of CFA/LPS and restimulated with the same antigen 1 week later before the proliferation **(E)**, and intracellular IFN-g levels **(F)** in tissue CD8^+^T cells in response to OVA antigen stimulation were compared between spleen and vascular tissues. Shown here are representative plots of experiments with at least three biological replicates. The data are shown as mean ± SEM (*n* = 3–8 mice) and analyzed using Student’s *t*-test. ^*^ *p* < 0.05; ^**^ *p* < 0.01; ^***^ *p* < 0.001. ns, no significant difference.

The activation of vascular DCs in response to systemic inflammation prompted us to inspect their functional impact on the tissue T cells immediately nearby. We first examined the expression levels of early activation marker CD69 on the vascular T cells *in situ* following LPS injection and found that vascular T cells had a higher expression and greater fold increase of CD69 over levels during the resting state when compared with that of splenic T cells (**Figures 5A, B**). This occurred in both vascular CD4^+^ and CD8^+^T cells, albeit the latter had higher levels of CD69 expression (**Figures 5C**, **6A**).

Next, we examined T-cell proliferation profiles *in situ via* Ki67 expression. We found that the percentage of proliferative vascular CD4^+^ and CD8^+^T cells was higher than their splenic counterparts after LPS stimulation (**Figures 5D**, **6B**). Interestingly, fold increase of proliferative vascular CD4^+^T cells after LPS over resting state was greater than that of CD8^+^T cells (**Figures 5E**, **6C**), indicating that CD4^+^T cells were more responsive to pathogen invasion in vascular tissues.

To confirm antigen-specific activation of T cells *in situ* by vascular DCs under inflammatory conditions, we injected ovalbumin (OVA) into the OT-II mice and measured the ability of vascular DCs to present the antigen for OVA-specific T stimulation. We found that vascular DCs were the prime APCs to present OVA peptide on MHC molecule in comparison with macrophages and B cells [(31, 32) and data not shown]. In this antigen-specific system mainly driven by the DCs, we found that there was a >4-fold increase in the proportion of Ki67^+^ vascular OT-II CD4^+^T cells in the presence of OVA compared with the absence of OVA under inflammatory conditions (**Figure 5F**). In the spleen, there was a more modest 2-fold increase between OVA and PBS injection (**Figure 5F**). Next, we determined to measure proliferation more directly by CFSE dilution. We observed that the proliferation of CD4^+^T cells in vascular tissues was greater than that of spleen (**Figure 5G**).

Since the density of DCs in vascular tissues is higher than that of spleen (**Figure 1B**), the elevated T-cell activity in the vascular tissues could be caused by better chance of encountering DCs by vascular T cells as compared with their splenic counterparts. To compare the qualitive difference of DCs and find out which subset stimulates T cells better in these two tissues, we sorted equal number of moDCs, cDC1 and cDC2 from WT mice, and cocultured with CFSE-labeled CD4^+^T cells from OT-II mice in the presence of OVA, respectively. We found that cDCs had generally higher capacity to induce proliferation of CD4^+^T cells than that of moDCs in both vascular tissues and spleens, in which splenic cDC2 is better than cDC1 as reported (33, 34); however, reverse trend was found within vascular cDC subsets (**Figure 5H**). Interestingly, between the tissues, vascular cDC1were much better at promoting CD4^+^T-cell proliferation than splenic cDC1, although vascular cDC2 were poorer than their splenic counterpart, suggesting better CD4^+^T-cell activation potential by vascular cDC1 per se (**Figure 5H**). These results identified vascular cDC1 as the main driver of the elevated CD4^+^T-cell proliferation in the vascular tissues *in vivo*.

In terms of cross-presentation of antigen, we found that vascular DCs presented more MHCI-OVA peptide complex *in vivo* than splenic DCs following LPS administration (**Figure 6D**). Within DC subsets, like their splenic counterparts, vascular cDC1 had the best capacity to cross-present MHCI-OVA peptide complex on single-cell levels (**Figure 6D**), indicating an outstanding potential of this vascular DC subset for CD8^+^T-cell stimulation. To confirm the functional outcomes of the *ex vivo* finding, we went on to compare the antigen-specific activities of CD8^+^ T cells *in vivo* between the two tissues in response to exogenous model antigen OVA. We found that following the OVA administration *in vivo*, vascular CD8^+^T cells responded by better proliferation (**Figure 6E**) and functionally produced much more IFN-g (**Figure 6F**) than splenic CD8^+^T cells did, indicating that the vascular CD8^+^T cells were more activated by exogenously cross-presented antigen.

### T-Cell Differentiation Corresponds With the Cytokine Profile of Vascular DCs *In Vivo* During Inflammation

**Figure 7:**
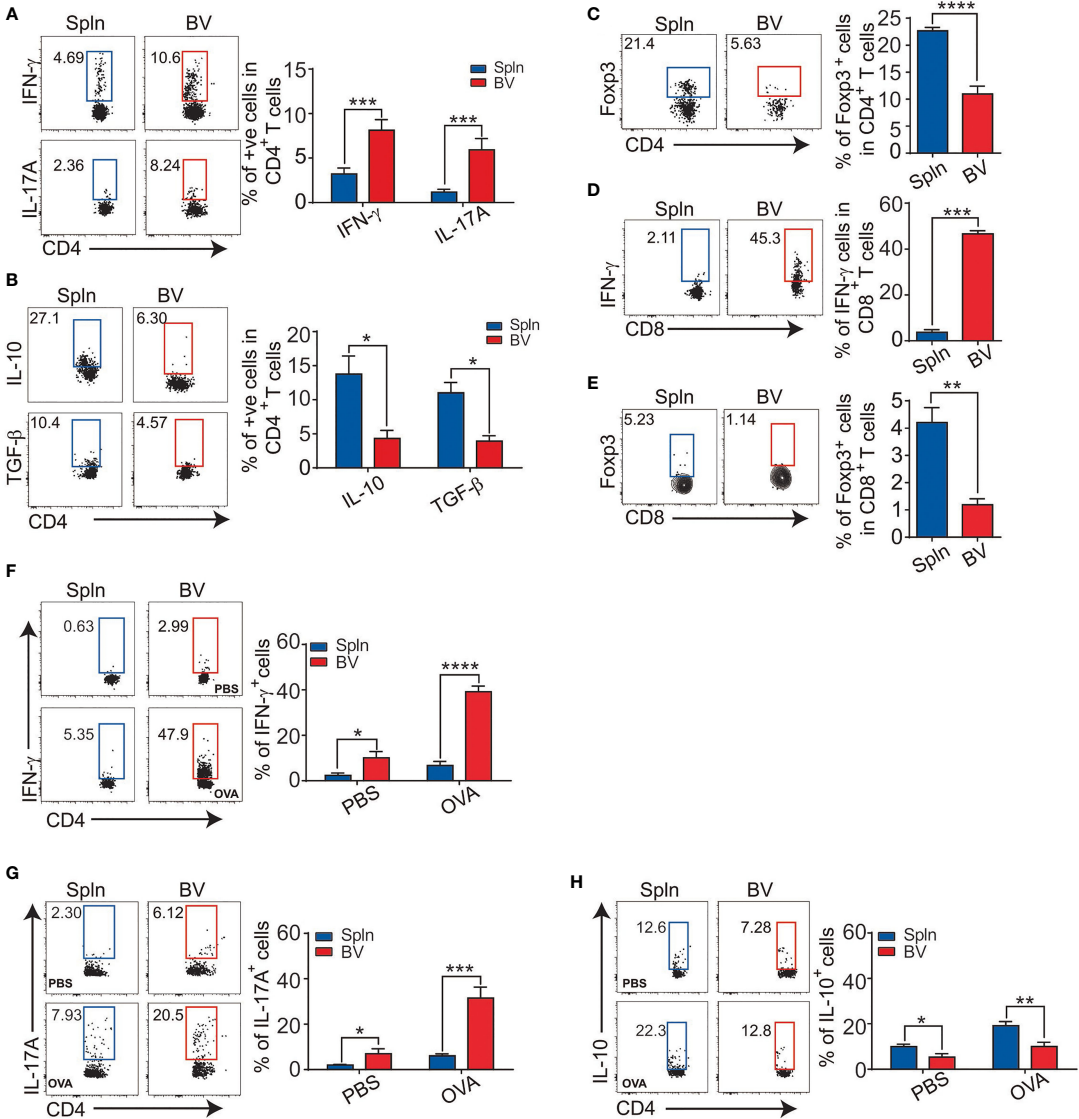
T-cell differentiation in vascular tissues during inflammation. Mice were injected with LPS before their BV and Spln were extracted to analyze the expression of IFN-γ, IL-17A **(A)**, IL-10, TGF-β1 **(B)**, and Foxp3 **(C)** in CD4^+^T cells and IFN-γ **(D)** and Foxp3 **(E)** in CD8^+^T cells. Alternatively, OT-II mice were injected with 200 µg OVA or PBS/LPS with LPS for 72 h before FACS analysis of indicated cytokines in CD4^+^T cells **(F–H)**. Shown here are representative plots of experiments with at least two biological replicates. The data are shown as mean ± SEM (*n* = 2–16 mice) and analyzed using Student’s *t*-test. ^*^ *p* < 0.05; ^**^ *p* < 0.01; ^***^ *p* < 0.001; ^****^ *p* < 0.0001.

To investigate the differentiation profiles of downstream effector cells of local DCs following their production of instructive cytokines, we first compared the signature cytokines of vascular and splenic T cells *in situ* in mice after LPS challenge. Consistent with the inflammatory polarizing cytokine secretion profiles of the vascular DCs, in which IL-12 for Th1, and IL-10 for Treg by moDCs, whereas IL-1 for Th17 by all DCs (**Figure 4**), a significantly higher proportion of vascular CD4^+^T cells than their splenic counterparts were differentiated into Th1, and Th17 effector cells based on their intracellular staining of IFN-γ and IL-17A, respectively (**Figure 7A**). In contrast, the proportion of vascular CD4^+^T cells expressing anti-inflammatory cytokines (IL-10 and TGF-β or Foxp3) was less than that of splenic CD4^+^T cells (**Figures 7B, C**). Concordant with the findings for CD4^+^T cells, the proportion of vascular CD8^+^T cells compared with that of splenic CD8^+^T cells, expressing IFN-γ was higher and expressing Foxp3 was lower (**Figures 7D, E**). We also tested these in an antigen-specific system by injecting OVA antigen *i.v.* into OT-II mice. After 72 h, we found a higher proportion of vascular CD4^+^T cells expressing IFN-γ, and IL-17A, but a lower proportion expressing IL-10 (**Figures 7F–H**), reinforcing a fundamental role of different vascular DC subsets in driving different local T-cell differentiation.

### Elevated B-Cell Activating Factors From Vascular moDCs Are Linked to the Active B-Cell Phenotypes in the Tissues

**Figure 8:**
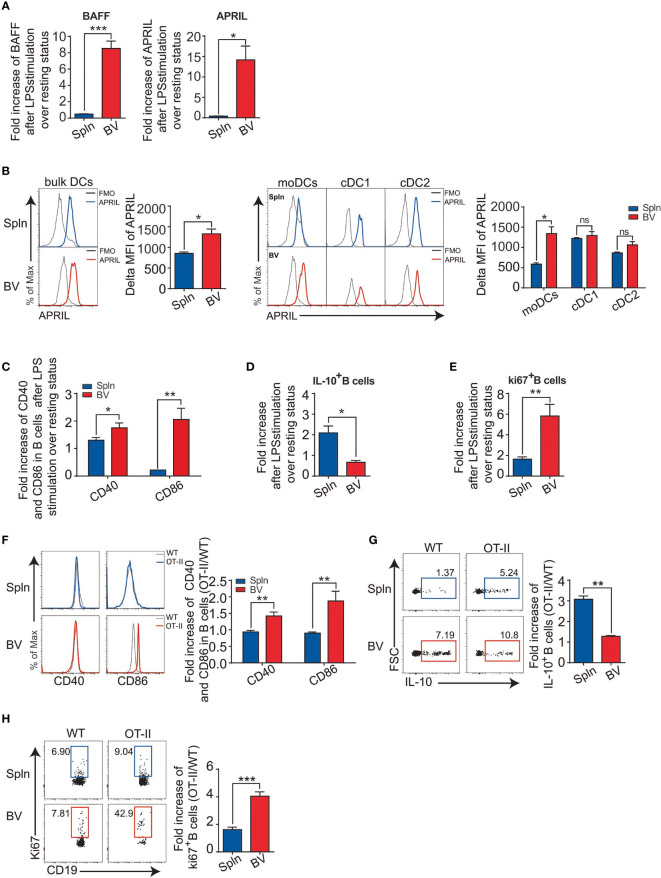
DC-derived B-cell activation factors and antigen-specific B-cell activation in vascular tissues and spleen. WT mice were *i.v.* injected with LPS or PBS before their BV and Spln were taken. Bar graphs showed the fold increase of BAFF and APRIL mRNA in the two tissues **(A)**; APRIL expression **(B)** on the DCs and their subsets from the two tissues; and costimulatory molecules CD40 and CD86 on B cells **(C)**, IL-10-producing B cells **(D)**, and proliferated B cells **(E)** from the two tissues. Shown here are representative plots of at least three biological replicates. Alternatively, WT and OT-II mice were *i.v.* injected with OVA/LPS before their BV and Spln were taken for FACS analysis of the fold increase of CD40/CD86 on B cells **(F)**, the percentage and fold increase of IL-10^+^B cells **(G)**, and the percentage and fold increase of Ki67^+^B cells **(H)** in OT-II over WT in BV and Spln. Shown here are representative plots of experiments with at least two biological replicates. The data are shown as mean ± SEM (*n* = 2–10 mice) and analyzed using Student’s *t*-test. ^*^ *p* < 0.05; ^**^ *p* < 0.01; ^***^ *p* < 0.001. ns, no significant difference.

In addition to cellular immunity, humoral immunity was also examined in vascular tissues following the acute inflammatory challenge. We found that despite proportionally fewer B cells (**Figure 1F**), the increase in B-cell activating factor (BAFF) and a proliferation-inducing ligand (APRIL) mRNA levels from LPS injection compared that the steady state was greater in vascular tissues than that in spleens (**Figure 8A**). Interestingly, we found that the expression of APRIL on vascular DCs was higher than that of splenic DCs, which was best represented by moDC, but not other cDCs (**Figure 8B**), indicating an outstanding role of vascular moDCs in B-cell activation. Consistent with this, it has been reported that LPS could induce moDCs to produce BAFF/APRIL and present antigen to activate B cells for plasma cell differentiation (35). Next, we measured the expression of costimulatory molecules in B cells and found that the increase in CD40 and CD86 induced by LPS was greater for vascular tissues than for spleens (**Figure 8C**). Interestingly, we also observed that the fold increase of IL-10-producing B cells in vascular tissues after LPS stimulation over the resting state was lower than that of spleens (**Figure 8D**), suggesting a reduced activity of regulatory B cells in the vascular tissues. Finally, we also detected a higher fold increase of Ki67^+^-expressing B cells by LPS stimulation in vascular tissues than that in spleens (**Figure 8E**), indicating that the capacity of vascular B-cell proliferation *in situ* is greater than their splenic counterparts in response to LPS administration. To identify the role of DCs, we employed OVA/OTII system. Concordantly, 72 h after OVA antigen were injected *i.v.* into OT-II mice, we also found that vascular B cells had higher levels of CD40/CD86 expression, lower IL-10 production, and more Ki67^+^B cells than that of spleens following the comparison with WT littermates (**Figures 8F–H**). Collectively, these data demonstrated an elevated activation of B-cell immunity induced by DCs in the vascular tissues following inflammatory stimulation.

### Establishment of More Severe Inflammation in Vascular Tissues by Systemic Inflammation With Increased Cells Accumulated in the Intima Region

**Figure 9:**
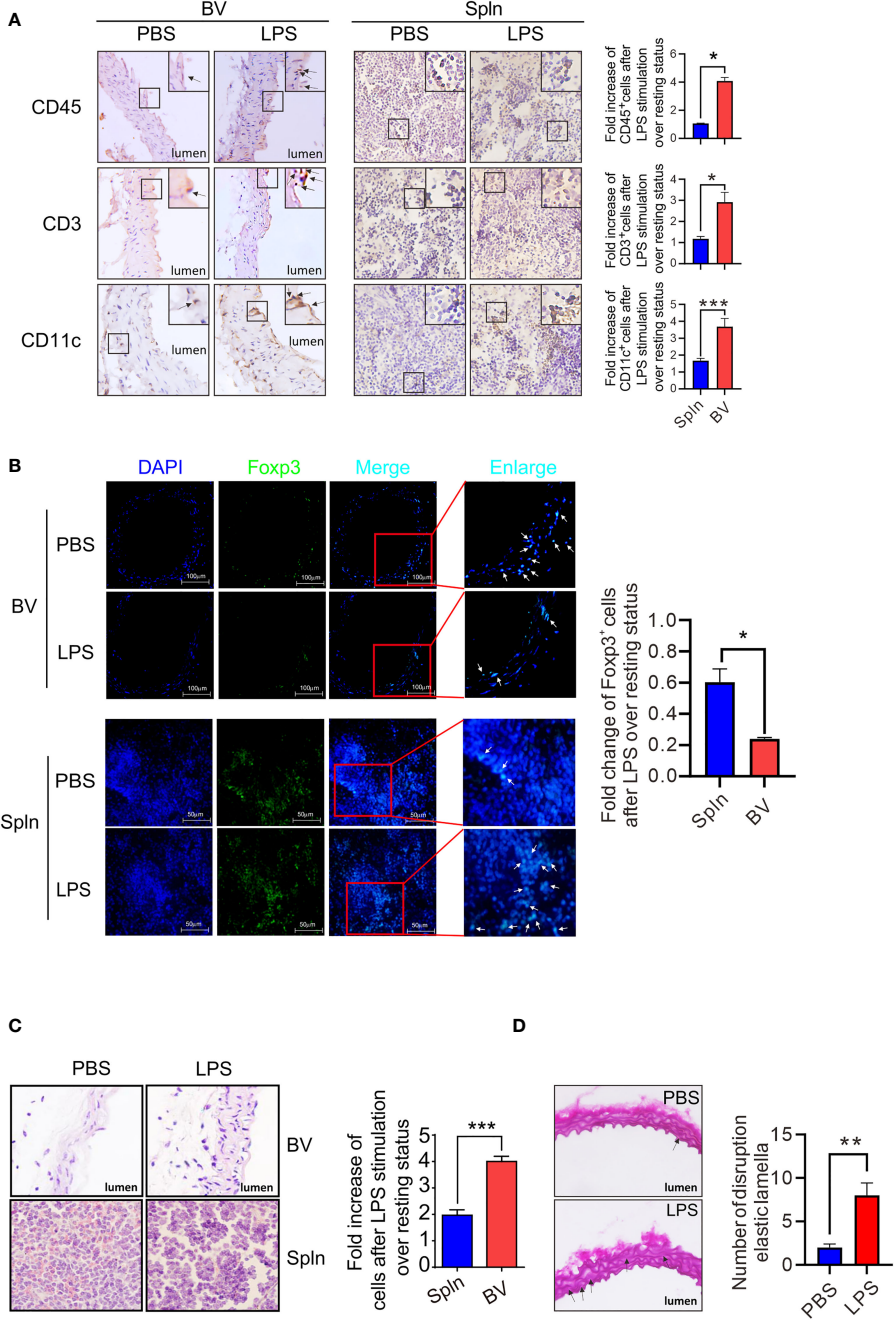
Establishment of vascular inflammation induced by systemic infection. Mice were *i.v.* injected with LPS or PBS before their BV and Spln were taken to analyze the expression of makers for leukocytes, T cells, and DCs by immunohistochemistry (magnification: ×40, with more magnified pictures for the representative positive cells at the top right boxes) **(A)**, Tregs by immunofluorescence (magnification: ×40) **(B)**, structure damages by HE staining in paraformaldehyde-fixed sections (magnification: ×100) **(C)**, and cross-sections of the aorta stained with Weigert coloration for elastic lamellae disruptions indicated by arrows (red: collagenous fiber; atropurpureus and blue: elastic fibers) (magnification: ×40) **(D)**. Shown here are representative plots of at least two experiments performed. The data are shown as mean ± SEM and analyzed using Student’s *t*-test. ^*^ *p* < 0.05; ^**^ *p* < 0.01; ^***^ *p* < 0.001.

We had shown above that the local immune cells could switch from a tolerogenic to activated state. Therefore, we wished to determine whether such activation could cause inflammation and tissue injury if not properly controlled. We first examined the distribution of the immune cells within the tissues before and after the induction of inflammation by immunohistochemistry staining and found that compared with normal/noninflamed mice, CD45^+^leukocytes, CD3^+^T cells, and CD11c^+^DCs increased in both vascular tissues and spleen, but the fold increase of these immune cells in vascular tissues were higher than that of the spleen (**Figure 9A**). Moreover, these cells in vascular tissues were observed to accumulate in the intima region following LPS administration (**Figure 9A**). Interestingly, in the vascular walls at resting states, T cells could be found in close proximity to DCs. After LPS stimulation, however, they formed interactions with each other (**Figure S3**). In addition, although a total number of T cells increased in the vascular tissues, Foxp3^+^Tregs decreased in response to LPS injection (**Figure 9B**), whereas Foxp3^+^Tregs in spleens increased under the same condition (**Figure 9B**). Finally, to verify the inflammatory outcomes of the activated local immunity to the vascular tissue, HE staining was performed on the longitudinal sections of vascular tissues and spleen from normal/noninflamed or LPS-injected inflamed mice. We found that after LPS treatment, immune cells increased in the vascular intima and some structural damages were visible, where medial and intimal areas were thicker than those of PBS controls (**Figure 9C**), and the presence of more disruptions of the elastic lamellae was apparent (**Figure 9D**).

## Discussion

In this study, we investigated regional immunological features of healthy blood vessel tissues. Through side-by-side comparison with systemic lymphoid organ spleen, we demonstrated that normal vascular tissues contained comprehensive immune cell types including both innate and adaptive components, indicating immunocompetency of the nonlymphoid circulatory tissues. Interestingly, at the steady state, moDCs, the most abundant DC subset in the vascular tissues, constitutively secreted suppressive cytokines and possessed superior capacity for antigen uptake, which is concordant with the view that vascular tissues may be immune privilege sites under physiological conditions. In response to inflammatory stimuli, however, this immunotolerance of vascular tissues was readily broken down by vascular DCs with a clear division of labor among subsets, demonstrating either enhanced proinflammatory cytokine, B-cell activating factor, but reduced anti-inflammatory cytokine expression by moDCs, or stronger immunogenic capacities to stimulate local CD4^+^ and CD8^+^ T by cDC1, which ultimately resulted in vascular autoinflammatory pathology.

More than a decade ago, normal healthy vascular tissues were found to contain a limited set of immune cells (36), of which CD11c^+^DCs, mostly resided in cardiac valves and aortic sinus, can capture blood-born OVA *in vivo* and present the antigenic peptide to stimulate CD8^+^T cells *in vitro* (31). Here, we have greatly expanded that study to identify more immune cell lineages and complete DC subsets in the normal vascular tissues. Our findings of relatively higher percentage of moDCs in vascular tissues with unique cytokine secretion, antigen uptake and B-cell activating profiles, and cDC1 with superior stimulatory competency to T cells *in situ* than lymphoid organ during inflammation indicated the importance of this antigen-presenting cells in regional immunity with multiple functionalities.

Because of their vital functions, the wall structures of medium and large arteries are immunoprivileged and protected from autoimmune destruction. This concept of immunoprivilege is not only limited to a series of cellular barriers that control immune cell entry but also extended to the development of tolerogenic immune cells in local microenvironment (37). Concordantly, we found that vascular DCs had a relatively immature phenotype compared with that of splenic DCs, and their expression levels of costimulatory molecules were lower, but coinhibitory molecule, PD-L1, were higher at the steady state, indicating their tolerogenic potential. Moreover, we found that the vascular DCs, particularly moDCs, constitutively secreted suppressive cytokines, IL-10, whereas little, if any, of the suppressive cytokine was produced by the splenic DCs. Along this line, a similar functional profile has been reported for hepatic DCs, and it has been proposed that liver tolerogenicity is related to specialized DCs (38). Given the gravity of blood vessel damage especially in medium and large vessels to the host, an immunological milieu towards a tolerogenic phenotype would provide an adaptable mechanism to protect vital vasculature from immune attack (39).

Immature DCs are better at taking up soluble and particulate material than mature DCs, and this quality enables immature DCs to present self-antigens to maintain self-tolerance (30). Likewise, we found that vascular DCs, especially moDCs, had superior capacity to phagocytose soluble antigens than their splenic counterparts did in the steady state. These results are quite interesting, as several autoantigens related to the atherosclerotic diseases have been identified such as oxLDL, HSP60, malondialdehyde-modified-LDL, β-galactosidase, etc. in the vascular walls (40–44). The improved antigen uptake capacity with immature phenotype of vascular DCs suggests that the DCs could possibly present these autoantigens during the steady state to maintain self-tolerance. To the best of our knowledge, this is the first demonstration of the uptake capacity of vascular DCs *in vivo*.

Although in a closed system, the vascular tissues can be invaded by foreign antigens from paralymphoid nodes or modification of autoantigen occurs during inflammation (42). Multiple papers have suggested that bacterial materials can be found in temporal artery sections (45, 46). Concordantly, we found that the immature state of arterial DCs could easily be overcome, and circulating bacterial compound LPS was sufficient to drive vascular DCs into maturation and reduce their inhibitive PD-L1 expression to prepare them to activate and differentiate local T and B cells for vascular tissue destruction, as we observed enhanced vascular T-cell proliferation *in situ* and differentiation towards inflammatory effector cells in response to LPS administration. Consistently, PD-L1 expression in vascular DCs was shown to be downregulated in human giant cell arteritis (GCA) (47, 48). Likewise, the inflammatory activity of vascular lesions in GCA was found to be driven by adaptive immune responses, where T cells undergoing clonal expansion (49) and their derived IFN-γ and local proinflammatory T-cell differentiation are closely correlated with clinical manifestations (50). Collectively, our data presented here would fit with an infection breaking the resting tolerogenic state of vascular DCs and initiating autoimmunity. Since vascular DCs were highly responsive to blood-borne pathogen-associated molecular patterns, such as LPS, for T-cell stimulation as demonstrated in the current study, the onset of vasculitis does not necessarily need to be induced by infection in the artery itself, so long as the pathogen-derived material enters the circulation.

It is now clear that human medium-sized arteries possess an indigenous DC population that critically contributes to regulating vascular wall inflammation (51). However, whether local infiltrated innate immune cells like macrophages or DCs function as APCs, initiating and sustaining activation of adaptive immune responses *in situ* in the vascular tissues, is far from clear. It has been reported that vessel wall-embedded DCs outperformed differentiated macrophages as APCs, whereas monocytes failed to upregulate essential surface receptors, such that they cannot promote intramural T-cell activation (32). Along this line, antibody-mediated global DC depletion was found to disrupt T-cell and macrophage activation and have marked anti-inflammatory effects in the vascular tissues (52). However, it remains controversial as to whether DCs can prime T cell within the local vascular tissues rather than migrating to SLOs to achieve this goal. In the current study, we found that vascular tissues contain naïve T cells (**Figure 3B**), and vascular resident DCs had constitutively higher expression of CCR7 (data now shown) that creates a scenario through binding to its cognate chemokines in which the highly activated DCs are trapped in the lesions and cannot migrate to the lymphoid tissues (53). To further demonstrate a DC-specific priming of local T cells *in situ* in our system, we utilized OVA/OTII system in the current study. The elevated vascular T-cell activation and proinflammatory differentiation indicated a strong potency of vascular DCs to stimulate local T cells in the specialized wall microenvironment. In support of this concept, DC-T cells were observed by confocal microscopy to engage in cell contact following antigen challenge (54) or LPS stimulation (**Figure S3**), providing visual evidence for naïve T-cell priming in vascular tissues.

Another important finding in our study is that most of the unique functions of vascular DCs were implemented by moDCs, as they comprise almost half of the bulk population (and thus, were the most abundant DC subset). Concordantly, BM-derived monocytes were found to be recruited to the normal aortic intima as the predominant mechanism for DC accumulation in the regions predisposed to atherosclerosis (55), and monocyte-derived resident intimal DCs efficiently ingest lipoprotein particles and rapidly transform into foam cells, earlier than recruited intimal macrophages and monocytes (56). Although we found vascular moDCs constitutively expressed inhibitive factors and were the major inducers of tissue tolerance, this particular DC subset quickly upregulates proinflammatory cytokines and B-cell activating factor partly because they express higher TLR4 to facilitate local T-cell differentiation and B-cell activation in response to systemic infection, whereas vascular cDC1 were more involved in presenting antigen peptide for both CD4^+^ and CD8^+^ T-cell proliferations, demonstrating a clear division of labor between DC subsets for therapeutic targeting. This concurs with the selective functions of moDCs and cDCs ascribed in SLOs (10, 28) but differs slightly with regional features.

It has become increasingly critical to decipher how organ physiology is integrated with resident immune cells during tissue development, health state, and disease state (57). Here, we described flow cytometry- and immunohistochemistry-based methods to study the composition as well as the functions of immune cells in healthy vascular tissues *in situ* and compared them with that of spleens in the C57BL/6 mice in the steady state. We revealed that the vascular immune cells, especially DCs possessed unique immunological features in both steady and inflammatory states. Therefore, we proposed that blood vessels, by virtue of their unique structure and microenvironment, have emerged as immunocompetent organs that are at risk to host-autoreactive inflammatory responses. A potential limitation of our study is that no vascular tissue-specific metabolic factor(s) was revealed for the observed regional immunological features. Further understanding of the intricacies of these integrated tissue-specific immune constituents with identification of contributing factors from local milieu in healthy individuals will help define the autoimmune mechanisms that impact inflammation and allow for more judicious therapeutic approaches to vascular autoinflammatory diseases.

## Data Availability Statement

The raw data supporting the conclusions of this article will be made available by the authors, without undue reservation.

## Ethics Statement

The animal study was reviewed and approved by the Institutional Animal Care and Use Committee of Anhui Normal University.

## Author Contributions

LS and WZ performed the experiments, made statistic studies, and analyzed the data. LZ, YZ, and FW helped perform the experiments. AL edited the manuscript. YX conceived the ideas, designed the experiments, analyzed the data, and wrote the manuscript. All authors have made a substantial, direct and intellectual contribution to the work, and approved it for publication.

## Funding

This work was financially supported by a National Natural Science Foundation Major Research Project, China (91742101); Anhui International Science and Technology Collaborative Project, China (1604b0602017); Anhui Natural Science Research Fund, China (1608085MH160); National Health and Medical Research Council grants, Australia (1143976, 1150425); Anhui Provincial Key Laboratory of Molecular Enzymology and Mechanism of Major Diseases; and Key Laboratory of Biomedicine in Gene Diseases and Health of Anhui Higher Education Institutes.

## Conflict of Interest

The authors declare that the research was conducted in the absence of any commercial or financial relationships that could be construed as a potential conflict of interest.

## Publisher’s Note

All claims expressed in this article are solely those of the authors and do not necessarily represent those of their affiliated organizations, or those of the publisher, the editors and the reviewers. Any product that may be evaluated in this article, or claim that may be made by its manufacturer, is not guaranteed or endorsed by the publisher.
