# Supplementary material for: Self-Tolerance of Vascular Tissues Is Broken Down by Vascular Dendritic Cells in Response to Systemic Inflammation to Initiate Regional Autoinflammation
Source: Front Immunol. 2022 Jan 26;13:823853. doi: 10.3389/fimmu.2022.823853 (PMC8825784; doi:10.3389/fimmu.2022.823853)
Supplement: Supplementary file 1 [file DataSheet_1.docx]

**SUPPLEMENTARY FIGURES**


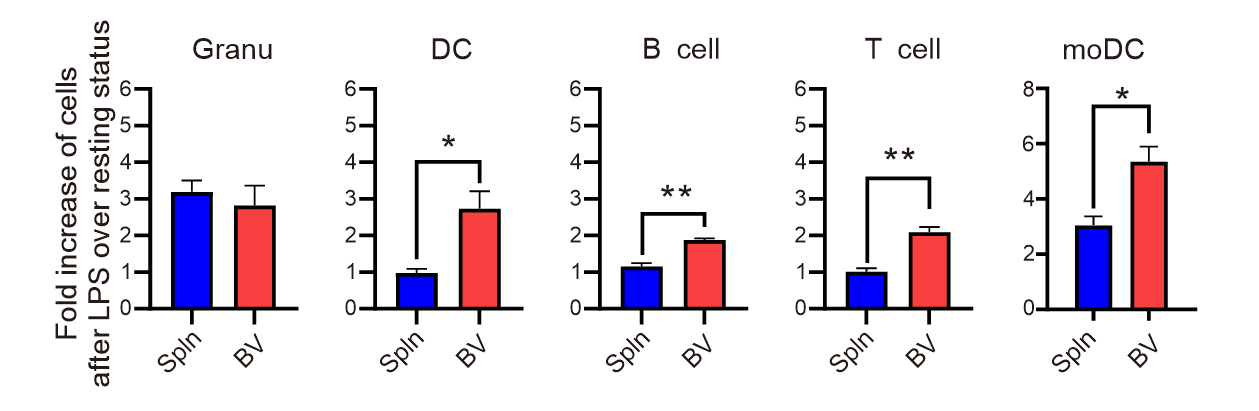


**Figure S1. The changes of immune cells in vascular tissues and spleens following inflammation.**

Mice were injected *i.v.* with LPS or not before their blood vascular tissues (BV) and spleens (Spln) were taken to make single cell suspension for FACS analysis. Bar graphs showed the fold increase of immune cells after LPS stimulation over resting status. The data were shown as Mean ± SEM (𝑛=3-4 mice), and analyzed using Student’s t test. *p<0.05, **p<0.01.


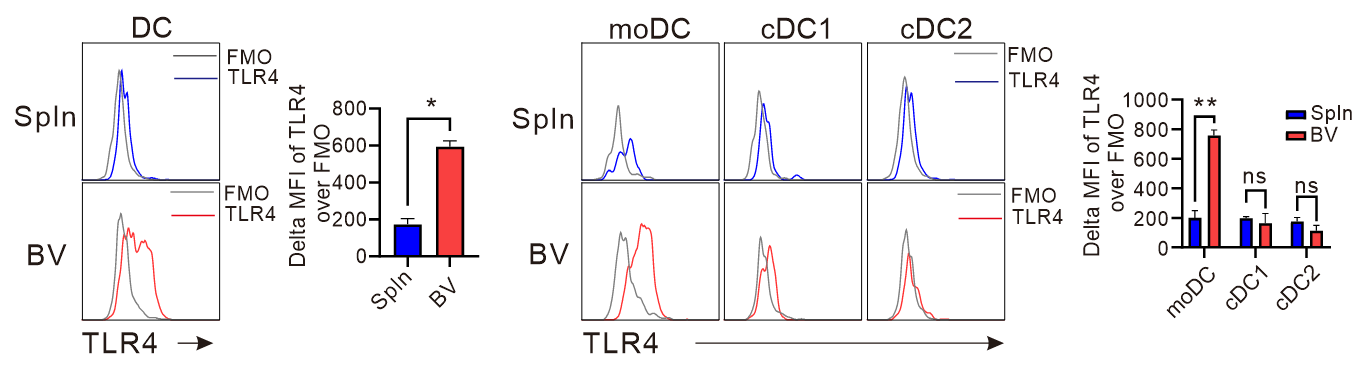


**Figure S2. The expression of TLR4 on DCs in vascular tissues and spleen.**

Single cell suspensions from BV and Spln of healthy/ noninflamed C57BL/6 mice were processed and stained for FACS analysis as before. The expression of TLR4 were demonstrated by delta MFI (MFI^TLR4^-MFI^FMO^). The data were shown as Mean ± SEM (𝑛=3-4 mice), and analyzed using Student’s t test. *p<0.05, **p<0.01.


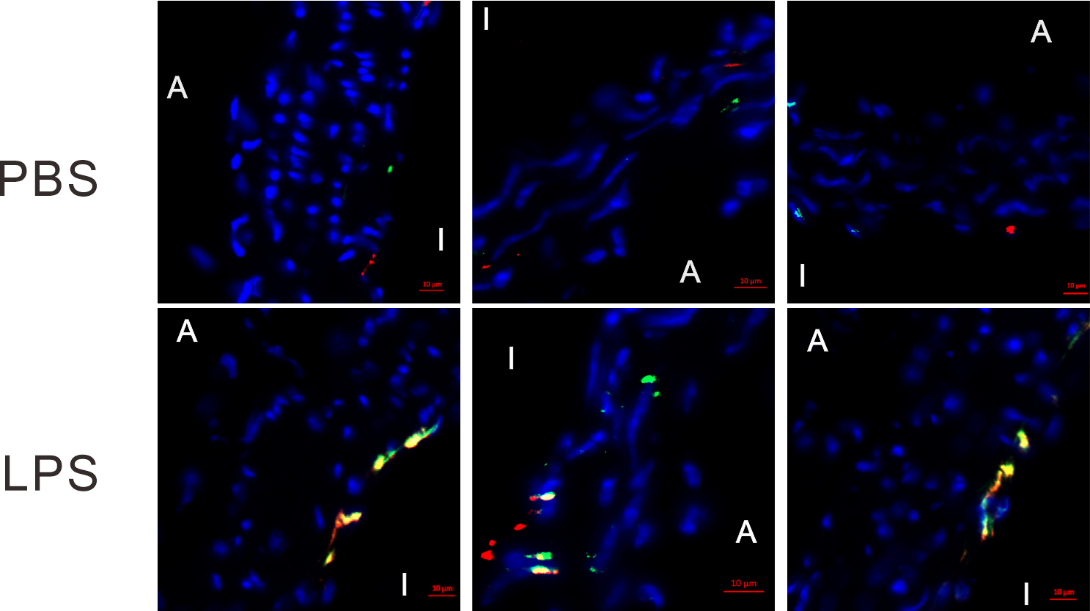


**Figure S3. Spatial relationship between DCs and T cells within the vascular compartments.**

Mice were injected with LPS or PBS before their aorta were taken. Longitudinal aortic sections were stained for immunofluorescence. Cell nuclei were stained with DAPI (blue). CD3^+^ T cells (red) and DCs (green) were found located in close proximity in vascular tissues of PBS treated samples (upper panel). In LPS treated mice, the number of T cells and DCs were both enriched and some of them formed interactions (yellow) close to the intima of vasculatures (lower panel). A indicates adventitia; I indicates intima. Scale bars = 10 μM.
